# Circadian Patterns of Intraocular Pressure Fluctuation among Normal-Tension Glaucoma Optic Disc Phenotypes

**DOI:** 10.1371/journal.pone.0168030

**Published:** 2016-12-13

**Authors:** Yeji Moon, Junki Kwon, Da Woon Jeong, Jin Young Lee, Jong Rak Lee, Seungbong Han, Michael S. Kook

**Affiliations:** 1 Department of Ophthalmology, University of Ulsan, College of Medicine, Asan Medical Center, Seoul, Korea; 2 Department of Applied Statistics, Gachon University, Seongnam-Si, Gyeonggi-do, Korea; Bascom Palmer Eye Institute, UNITED STATES

## Abstract

**Objective:**

To characterize the 24-h habitual-position intraocular pressure (IOP) patterns of optic disc phenotypes (ODPs) in untreated normal-tension glaucoma (NTG) and the relationships between nocturnal IOP elevation and various clinical factors.

**Design:**

Prospective, cross-sectional, observational study.

**Methods:**

Eighty-two NTG patients with focal ischemic (FI) ODP and 82 age- and disease severity-matched NTG patients with myopic glaucomatous (MG) ODP were recruited prospectively over 3 years. The IOP was recorded 11 times over a 24-hour (h) period by a single ophthalmologist using a hand-held tonometer (TonoPen^®^XL). A cosinor model was used to describe the 24-h IOP rhythm. Associations between nocturnal IOP elevation and both ocular and demographic variables were evaluated using the generalized estimating equation (GEE).

**Results:**

Mean habitual-position IOP was significantly higher during nighttime than daytime in the FI group (16.44 vs. 14.23 mmHg, P < 0.001), but not in the MG group (15.91 vs. 15.70 mmHg, P = 0.82). The FI group also exhibited a significantly higher peak IOP during sleeping hours (P = 0.01) and lower trough IOP during the 24-h period than the MG group (P < 0.01). The MG group showed a significantly higher peak IOP during waking hours than the FI group (P < 0.01). Therefore, 24-h IOP fluctuation range was significantly higher in the FI group than the MG group (P = 0.013). In the FI group, peak habitual-position IOP and the highest frequency of IOP peaks occurred during sleeping hours (12 AM–6 AM). By contrast, IOP peaks in the MG group occurred during morning hours (8 AM–12 PM). The FI group showed an overall nocturnal acrophase in habitual-position IOP, with 45 patients (54.9%) having a nocturnal acrophase; 10 (12.2%), a diurnal acrophase; and 27 (32.9%), no evident acrophase. By contrast, the MG group showed no evident peak in habitual-position IOP, with 9 patients (10.9%) having a nocturnal acrophase; 43 (52.4%), a diurnal acrophase; and 30 (36.6%), no evident acrophase. In multivariate modeling using the GEE, ODP (P < 0.001) and spherical equivalent (SE, P *=* 0.001) were independently associated with nocturnal IOP elevation.

**Conclusions:**

Based on 24-h habitual-position IOP data, FI is associated with significant nocturnal IOP elevation, while no such nocturnal IOP elevation is observed in MG ODP. In untreated NTG, there are also significant differences in the 24-h IOP pattern between FI and MG ODPs.

## Introduction

Untreated open-angle glaucoma (OAG) patients have a higher nocturnal (supine) intraocular pressure (IOP) than diurnal (seated) IOP in the habitual body position [1–3]. Thus, IOP measurements during daytime office hours may not be representative of IOP status at night. Studies including our recent work have suggested that increased 24-hour (h) IOP fluctuation due to elevated nocturnal IOP may be a risk factor for glaucomatous optic nerve head (ONH) and/or visual field (VF) damage [2–4]. Therefore, 24-h habitual-position IOP measurements to detect nocturnal IOP elevation in suspected and confirmed OAG patients are critical to enhance our knowledge of glaucoma pathogenesis and clinical management.

Four distinct optic disc phenotypes (ODPs) have been recognized in the glaucomatous ONH among OAG patients: focal ischemic (FI), myopic glaucomatous (MG), senile sclerotic (SS), and concentric enlargement (CE) [5]. Different 24-h IOP patterns may exist among OAG patients with different OPDs. For example, Deokule et al [6] reported that untreated primary open-angle glaucoma (POAG) patients with CE ODP showed higher mean IOP and a greater number of IOP peaks in the nocturnal period compared to those with non-CE ODPs, suggesting that ODP may be related to different aqueous humor dynamics (AHDs) that determine 24-h IOP pattern.

Different types of OAG show regional predilection. Several population-based studies in East Asia have reported that the majority of OAG subjects have normal-tension glaucoma (NTG) with an IOP in the “normal” statistical range [7, 8]. Recent studies have shown that MG is now the most common ODP in Asian NTG patients, as the prevalence of myopic refractive error in Asians has increased greatly over the past several decades [9–15]. Clinically, NTG often manifests first with VF defect in one hemifield associated with an OHN focal notch with or without retinal nerve fiber layer (RNFL) defects [16–18]. Following MG, FI ODP is the second most common form of ODPs found in NTG [14].

To the best of our knowledge, no study has evaluated the relationship between 24-h habitual-position IOP pattern and ODP in NTG patients. The purposes of the present study were to (i) compare the 24-h habitual-position IOP profiles, including peak IOP timing (acrophase), in matched groups of untreated NTG patients with FI and MG ODPs, and (ii) identify clinical factors (including ODP) associated with nocturnal IOP elevation in untreated NTG patients.

## Materials and Methods

### Patients

All NTG patients examined by a single glaucoma specialist (MSK) from March 2012 to September 2015 at the glaucoma clinic of the Asan Medical Center, Seoul, Korea, were prospectively and consecutively recruited. All procedures conformed to the Declaration of Helsinki, and the study was approved by the Institutional Review Board of Asan Medical Center at the University of Ulsan, Seoul, Korea. All patients provided written informed consent. All eligible NTG patients had glaucomatous ONHs with diffuse or focal neural rim thinning, disc hemorrhage, or retinal nerve fiber layer (RNFL) defects, along with corresponding glaucomatous VF loss on repeated examination. These patients showed best-corrected visual acuity (BCVA) > 20/40, untreated IOP ≤ 21 mmHg in both eyes at 8 AM, 12 PM, and 4 PM on the same day in the clinic using Goldmann applanation tonometry (GAT), and normal anterior chamber and open angle based on slit-lamp and gonioscopic examinations, respectively.

Glaucomatous VF defects were defined as those having two or more of the following criteria as confirmed by more than one reliable consecutive test in addition to compatibility with ONH appearance: (1) a cluster of three points with a probability of less than 5% on a pattern deviation (PD) map in at least one hemifield and including at least one point with a probability of less than 1%, (2) a glaucoma hemifield test (GHT) result outside 99% of the age-specific normal limit, and (3) a pattern standard deviation (PSD) outside 95% of the normal limit [2–4]. Reliable VF assessment was defined as a VF test with a false-positive error < 15%, a false-negative error < 15%, and a fixation loss < 20%. The second VF test was performed to confirm glaucoma within 2 to 4 weeks after the initial glaucomatous perimetry exam to control for learning effects [2–4].

Exclusion criteria included one or more of the following conditions: (i) untreated IOP ≥ 22 mmHg at 8 AM, 12 PM, and 4 PM on the same day during clinic hours in at least one eye as determined using GAT, (ii) previous or current use of systemic or topical antiglaucoma medications or steroids, (iii) history of massive systemic hemorrhage or hemodynamic crisis, (iv) evidence of intracranial or otolaryngological lesions, (v) presence of other ophthalmic disease that may result in ONH and VF defects such as diabetic retinopathy, or (vi) any ophthalmic disease other than glaucoma and mild cataracts. Individuals with an irregular daily sleep schedule and smokers and were also excluded, as were patients who had previous ocular surgeries (including refractive surgery) or corneal abnormalities as these conditions may prevent reliable IOP measurement. Finally, patients taking antihypertensive or other hemodynamically active medications were excluded from the current study. The affected eye was selected for patients with unilateral disease. If both eyes were diagnosed with NTG and met the inclusion criteria, one eye was selected randomly for the study.

An average central corneal thickness (CCT) was calculated based on 3 measurements at the initial visit for each patient using ultrasonic pachymetry (DGH-550, DGH Technology Inc., Exton, PA, USA). All procedures conformed to the Declaration of Helsinki, and the study was approved by the Institutional Review Board of Asan Medical Center at the University of Ulsan, Seoul, Korea. All patients provided informed consent.

### In-hospital 24-hour IOP Measurement

A single ophthalmologist (D.W.J.) performed all 24-h IOP measurements. Prior to this study, a separate study [19] to test the accuracy of the hand-held tonometer (TonoPen^®^XL, Mentor Ophthalmics, Santa Barbara, CA, USA) against GAT was performed by D.W.J. on 52 consecutive patients (104 eyes) with glaucoma or suspected glaucoma. Results showed a strong correlation between the 2 tonometer readings (r = 0.93, P < 0.001), with a difference of less than 2 mmHg in 95% of measurements.

The method of in-hospital 24-h IOP measurement has been extensively described in our previous studies [2,4,19,20]. Briefly, all eligible patients in the current study avoided alcohol and caffeine intake for 3 days prior to hospital admission. Using the TonoPen^®^XL, IOP was measured at 8 AM, 10 AM, 12 PM, 2 PM, 4 PM, 6 PM, 8 PM, and 10 PM (diurnal IOP), and at 12 AM, 3 AM, and 6 AM (nocturnal IOP) in both eyes. Prior to each IOP measurement, one or two drops of 0.5% proparacaine were used as topical anesthetic. At each time point above, three IOP measurements were obtained, and the average value was used for analysis. Subjects were instructed to continue normal indoor activities during the diurnal period, and diurnal IOP was measured when patients were seated in the upright position. At 10 PM, the nurse turned off the lights in individual rooms and patients slept with their head at the same level as their body. Nocturnal IOP measurements were obtained under dim light, with patients first in the supine position to estimate long-term IOP change from diurnal IOP (as nocturnal IOPs were measured following hours of sleep in the supine position). Additional IOP measurements were then performed in the upright position after a 10-min break to estimate short-term IOP changes (as IOP was measured following 10 minutes of posture change from supine to upright position). The IOP measures were not corrected for CCT.

### Classification of Optic Disc Phenotypes

**Fig 1:**
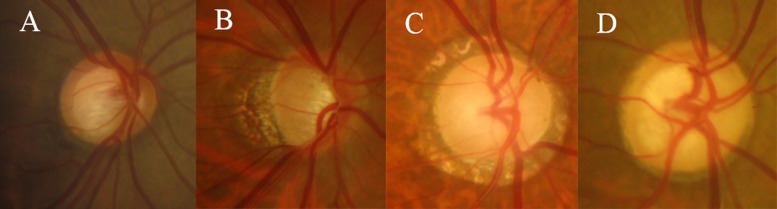
Photographs of four distinct optic disc phenotypes. (a) Focal ischemic optic disc, (b) Myopic optic disc, (c) Senile sclerotic optic disc, (d) Concentric enlarged optic disc.

As previously described in detail [14], the optic discs of all eligible NTG patients that underwent in-hospital 24-h IOP measurement were evaluated from stereoscopic optic disc photographs using a stereoscopic viewer (Pentax, Asahi, Japan). Two experienced glaucoma specialists (J.R.L. and M.S.K.) independently classified optic discs into one of the following ODP categories according to the method of Nicolela and Drance (Fig 1) [5]: (1) FI with localized neuroretinal rim loss (< 2 clock) at the superior pole, inferior pole, or both, but good preservation of the remaining neuroretinal rim, (2) MG with tilted optic discs showing a temporal crescent of additional glaucomatous damage characterized by neuroretinal rim thinning superiorly, inferiorly, temporally, or a combination thereof in the absence of degenerative myopia, (3) SS with a saucerized and shallow cup exhibiting a relatively pale, moth-eaten neuroretinal rim, parapapillary atrophy, and choroidal sclerosis, or (4) CE with enlarged round cups but no localized neuroretinal rim loss or pallor, and well preserved parapapillary retina.

Optic disc with a mixed ODP appearance was classified into one of the four groups according to the predominant feature. In the previous study, the same observers (J.R.L. and M.S.K.) agreed on 69 of 73 eyes (94.5%) during the first assessment using different sets of optic disc photographs of glaucomatous eyes [14]. As a result, the interobserver agreement yielded a κ value of 0.91 (excellent agreement). In the current study, if the two investigators (J.R.L. and M.S.K.) did not agree on initial classification, ODP assessment was made based on the consensus following a second review. If the observers could not reach a consensus, the patient was excluded from the final analysis. Both observers were masked to the 24-h IOP data, clinical information, and VF status of each optic disc evaluated. In the current study, only FI and MG ODPs were included in the analysis as these constitute the two most common ONH phenotypes in NTG patients [13–15] and the underlying pathogenesis is thought to differ between FI and MG ODPs [5, 6, 21]. Eyes with MG ODP were matched to those with FI ODP for age (≤ 5 years) and glaucoma severity (mean deviation [MD] ±1 dB) as these parameters may affect the 24-h IOP pattern.

## Statistical analysis

Based on our previous study [19] showing that OAG eyes with emmetropic refractive error (RE) (spherical equivalent [SE] > −3 D) had an average nocturnal IOP elevation > 1 mmHg with a standard deviation (SD) of 2.00 mmHg in the supine position, while those with myopic RE (SE ≤ −3.00 D) had no IOP elevation at nighttime, we hypothesized that the FI group would show greater nocturnal IOP elevation (>1.0 mm Hg) than the MG group in the supine position. A sample size of at least 52 subjects was needed for 80% power to detect a group difference in nocturnal IOP greater than 1.0 mmHg with a type Ι error probability of 0.05 in a two-tailed test assuming a SD of 1.75 mmHg.

Demographic and ocular baseline characteristics are expressed as number (%) or mean ± standard deviation as appropriate. Normally distributed numerical data were compared by independent samples t test and non-Gaussian data by Wilcoxon rank sum test. Categorical data were compared by chi-square test (FI vs. MG).

Generalized estimating equations (GEEs) are useful for fitting data obtained from clustered or repeated observations such as diurnal, nocturnal, and 24-h IOP measurements [22]. Therefore, GEEs were used to evaluate differences in habitual-position mean IOPs obtained at different time points (diurnal vs. nocturnal period) for each group. Various habitual-position IOP parameters, separated by different time periods, and the two posture-induced IOP changes (short-term and long-term) were compared between the two groups (FI vs. MG group) using GEEs [22]. Finally, measurements of mean peak diurnal and nocturnal IOP values, trough IOP (any time within 24-h), and peak minus trough (24-h range) IOP in the habitual position were compared between the two groups by paired *t*-test. We also evaluated the 24-h mean IOP flow and frequency of peak IOP values for each group.

We used least-squares cosinor rhythmometry to describe the 24-h habitual-position IOP pattern and acrophase in the current study. This model has been used extensively in previous studies to describe symmetric and stationary rhythmic patterns such as 24-h IOP behavior [2–4,19,20,23,24]. As described in our recent publications [2,4,19,20], the cosinor model is described as Y (t) = b0 + b1 × Cos[(2π/24) × t] + b2 × Sin[(2π/24) × t], where y represents the IOP at time t after the IOP measurement is initiated, and b0, b1, and b2 indicate regression coefficients. The constant (2π/24) and the coefficient b0 represent the 24-h periodicity of IOP and the 24-h rhythm-adjusted mean IOP, respectively. Using the cosinor model, 24-h IOP pattern was determined for each group and individual to classify patterns based on the acrophase (nocturnal, diurnal, and no acrophase). The acrophase distributions according to individual pattern were compared between the FI and MG group using the chi-squared test.

The nocturnal supine average IOP minus day sitting average IOP during 24 h was used to represent the nocturnal IOP elevation in the current study. As the GEE methods examines the associations between a repeatedly measured outcome variable (nocturnal IOP elevation) and dynamic and static predictors, the correlations between nocturnal IOP elevation and the various demographic and ocular variables were analyzed using GEE univariate and multivariate analyses [20,22]. The predictor variables included age, sex (reference: female), SE, CCT, pattern standard deviation (PSD), mean clinic IOP, body mass index (BMI), and ODP (reference: MG ODP). Following univariate analyses, variables with P < 0.2 were subsequently included in the multivariate analyses [20, 25]. These variables were combined in a single model to assess their effects on the nocturnal IOP elevation, while the joint effects of the related parameters on nocturnal IOP elevation were adjusted using GEE multivariate analysis [20, 25]. All statistical tests were performed using SPSS 15.0 for Windows (SPSS Inc., Chicago, IL). The criterion for statistical significance was P < 0.05.

## Results

A total of 188 eyes from 188 NTG subjects met the initial inclusion criteria for this study. Twenty-four eyes (12.8%) were excluded because the optic disc was considered non-classifiable, either because of poor photograph quality (n = 14) or lack of consensus due to mixed appearance (n = 10). A total of 164 eyes from 164 NTG patients were included in the final analysis, with 82 eyes categorized as FI and 82 eyes as MG. All subjects were native Koreans. Demographic and clinical characteristics of FI and MG OPD groups are summarized in Table 1.

**Table 1:** Demographic and ocular characteristics of the focal ischemic group and the myopic glaucomatous group.

Demography	FI group (n = 82)	MG group (n = 82)	P value
**Age (year)**			
Mean ± SD	51.72 ± 7.40	49.17 ± 7.11	0.29
Range	40–86	40–65	
**Sex (M/F)**	40/42	45/37	0.51
**SE (D)**			
Mean ± SD	-0.28 ± 1.76	-4.92 ± 2.25	<0.001
Range	-2.00 − 2.88	-8.25–-0.63	
**CCT (um)**			
Mean ± SD	548.41 ± 30.73	551.48 ± 41.22	0.83
Range	447.00–609.67	441.67–666.67	
**Humphrey VF, MD**			
Mean ± SD	-2.74 ± 3.52	-3.64 ± 5.15	0.18
Range	-15.17–2.20	-21.11–2.10	
**Humphrey VF, PSD**			
Mean ± SD	4.22 ± 3.77	4.43 ± 4.02	0.76
Range	1.10–16.80	1.10–15.70	
**Mean clinic IOP, GAT**			
Mean ± SD	14.33 ± 2.64	15.47 ± 2.04	0.004
Range	8.50–21.00	11.50–21.00	
**BMI (kg/m**^**2**^**)**			
Mean ± SD	23.75 ± 2.82	24.54 ± 3.13	0.10
Range	19.21–34.00	18.31–32.78	

FI, focal ischemic; MG, myopic glaucomatous; M, male; F, female; SE, spherical error; CCT, central corneal thickness; MD, mean deviation; PSD, pattern standard deviation; BMI, body mass index; SD, standard deviation;VF, visual field

There were no statistically significant group differences in age, sex ratio, CCT, MD, PSD, and BMI (P > 0.05).

Mean habitual-position IOP was significantly higher during nocturnal period than diurnal period in the FI group, (P < 0.01, Table 2); however, no such difference was found in the MG group (P = 0.82, Table 2). Mean habitual-position IOP during diurnal period was significantly higher in the MG group than the FI group (P < 0.01, Table 3), while mean habitual-position IOP during nocturnal period was higher in the FI group than the MG group, although the difference did not reach statistical significance (P = 0.34, Table 3). Both short- and long-term posture-induced IOP changes were significantly greater in the FI group than the MG group (P < 0.01 and P < 0.01, respectively, Table 4).

**Table 2:** Difference in mean habitual-position IOP at different times in each group.

	Daytime	Nighttime	P value
FI group	14.23 ± 2.13	16.44 ± 2.21	<0.01*
MG group	15.70 ± 2.22	15.91 ± 2.23	0.82

FI, focal ischemic; MG, myopic glaucomatous

* Statistically significant.

**Table 3:** Difference in mean habitual-position IOP during daytime and nighttime between the two groups.

	FI group	MG group	P value
Daytime, sitting	14.23 ± 2.1	15.70 ± 2.22	<0.01*
Nighttime, supine	16.44 ± 2.21	15.91 ± 2.23	0.34

FI, focal ischemic; MG, myopic glaucomatous

* Statistically significant.

**Table 4:** Difference in short-and long-term mean habitual-position IOP change between the two groups.

	FI group	MG group	P value
Night supine minus night sitting IOP (short-term)	2.38 ± 1.12	0.89 ± 1.01	<0.01*
Night supine minus day sitting IOP (long-term)	2.21 ± 0.88	0.21 ± 0.22	<0.01*

FI, focal ischemic; MG, myopic glaucomatous; IOP, intraocular pressure

* Statistically significant.

The MG group showed a significantly higher peak IOP during diurnal period than the FI group (P < 0.01). Conversely, the FI group showed a significantly higher peak IOP during nocturnal period (P = 0.01) as well as a lower trough IOP during the 24-h period than the MG group (P < 0.01). Therefore, the 24-h IOP fluctuation range (peak minus trough) was higher in the FI group than the MG group (P = 0.013) (Table 5).

**Table 5:** Difference in mean peak (day, night), trough (24 h), and peak-trough (24 h) IOP in the habitual position as measured by hand-held tonometer between the two groups.

	FI group	MG group	P value
Peak sitting (day)	15.03 ± 2.60	17.24 ± 2.99	<0.01
Peak supine (night)	17.23 ± 2.74	16.16 ± 2.25	0.01*
Trough sitting (24 h)	13.16 ± 2.26	15.11 ± 1.86	<0.01
Peak minus trough (24 h)	4.07 ± 1.68	2.13 ± 2.15	0.013*

FI, focal ischemic; MG, myopic glaucomatous

* Statistically significant.

**Fig 2:**
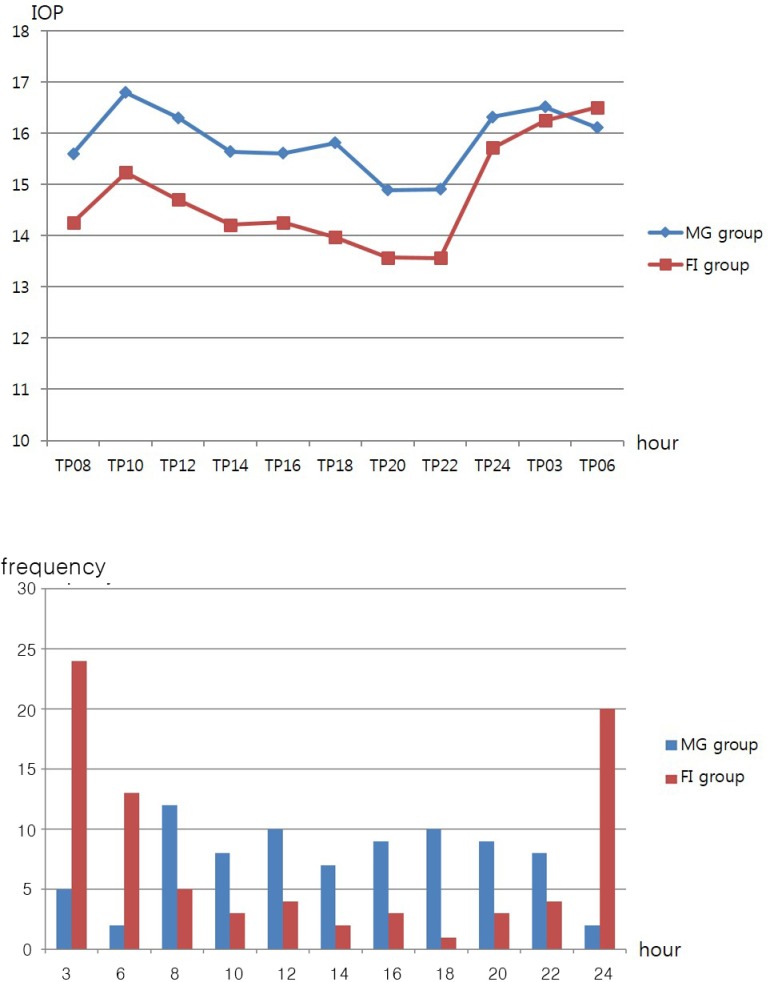
Comparison of 24-h mean IOP flow (A) and peak time frequency analysis of IOP (B).

Peak habitual-position IOP values and the highest frequency of IOP peaks occurred during morning hours (8 AM–12 PM) in the MG group (Fig 2A and 2B). By contrast, peak habitual-position IOP values and the highest frequency of IOP peaks occurred during sleeping hours (12 AM–6 AM) in the FI group (Fig 2A and 2B).

**Fig 3:**
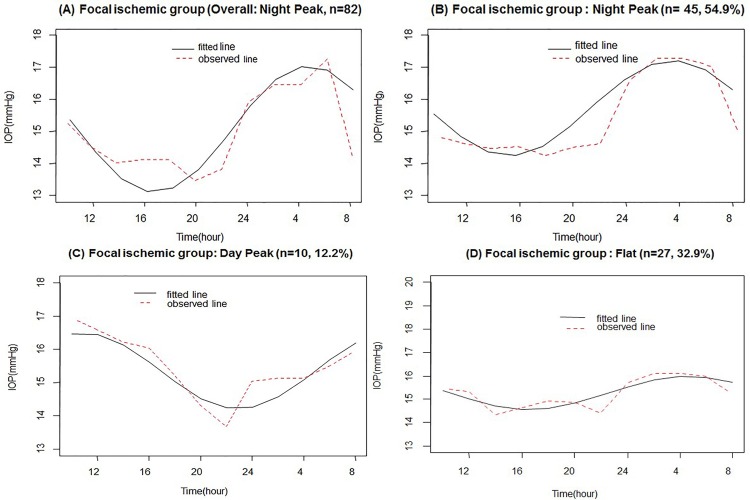
Average 24-h of habitual-position IOP rhythms in all patients (A) and in three subgroups of patients (B, C, and D) based on the cosinor model in the focal ischemic (FI) group.

A nocturnal peak (acrophase) of habitual-position IOP was found at 3–6 AM in the entire FI group based on the cosinor model during the 24-h period (Fig 3A). In an analysis of individual FI patients, 45 (54.9%) had a nocturnal acrophase (Fig 3B), 10 (12.2%) a diurnal acrophase (Fig 3C), and 27 (32.9%) no evident acrophase (Fig 3D).

**Fig 4:**
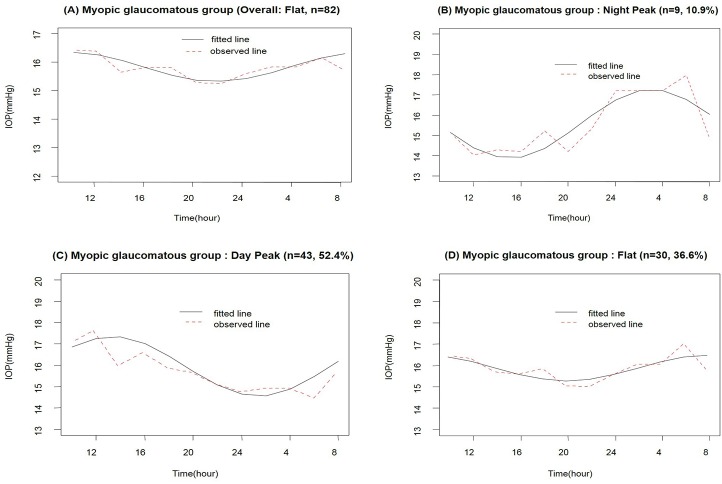
Average 24-h rhythms of habitual-position IOP in all patients (A) and in three subgroups of patients (B, C, and D) based on the cosinor model in the myopic glaucomatous group.

No evident peak (acrophase) in habitual-position IOP measurements was noted in an analysis of the entire MG group based on the cosinor model during the 24-h period (Fig 4A). However, analysis of individual MG patients indicated that 9 (10.9%) had a nocturnal acrophase (Fig 4B), 43 (52.4%) a diurnal acrophase (Fig 4C), and 30 (36.6%) no evident acrophase (Fig 4D).

Consequently, there were significant differences in the distribution of diurnal and nocturnal acrophases between the FI and MG group (P < 0.05, chi-squared test, Table 6).

**Table 6:** Difference in nocturnal and diurnal IOP patterns based on the cosinor model between the two groups.

	FI group	MG group	P value
Night peak	45 (54.9%)	9 (10.9%)	<0.01*
Day peak	10 (12.2%)	43 (52.4%)	0.01*
Flat	27 (32.9%)	30 (36.6%)	0.59
Total	82	82	

FI, focal ischemic; MG, myopic glaucomatous

* P < 0.05 (chi-squared test).

The results of univariate and multivariate modeling of the various clinical variables to predict nocturnal habitual-position IOP elevation (nocturnal supine average IOP minus diurnal sitting average IOP) are presented in Tables 7 and 8.

**Table 7:** Univariate Model for the Relationship between Nocturnal IOP Elevation and various Dynamic and Static Variables using Generalized Estimating Equation.

	Nocturnal IOP elevation	
Parameters	Estimated coefficient (beta)	P value
age	0.162	0.124
gender (reference: female)	-0.024	0.430
SE	0.361	0.023
CCT	0.023	0.171
MD	-0.004	0.901
PSD	0.031	0.750
BMI	0.053	0.464
office mean IOP	-0.063	0.431
ODP (reference: MG)	3.824	<0.001

Abbreviations: IOP, intraocular pressure; SE, spherical equivalent; CCT, central corneal thickness; MD, mean deviation; PSD, pattern standard deviation; BMI, body mass index; ODP, optic disc phenotype; MG, myopic glaucomatous

**Table 8:** Multivariate Model for the Relationship between Nocturnal IOP Elevation and various Dynamic and Static Variables using Generalized Estimating Equation.

	Nocturnal IOP elevation	
Parameters	Estimated coefficient	P value
age	0.162	0.124
SE	0.324	0.001
CCT	0.003	0.271
ODP (reference: MG)	3.35	<0.001

Abbreviations: IOP, intraocular pressure; SE, spherical equivalent; CCT, central corneal thickness; ODP, optic disc phenotype; MG, myopic glaucomatous

All variables with P value < 0.2 in univariate analysis were included in the GEE multivariate analysis in order to assess their joint effects on nocturnal IOP elevation. In the multivariate model, SE (P *=* 0.001) and ODP (P < 0.001) were found to be significantly associated with nocturnal IOP elevation (Table 8).

## Discussion

Different ODPs of glaucomatous ONHs have been found to be associated with distinct clinical characteristics that may be involved in glaucoma pathogenesis. For example, female patients and those with systemic vascular dysregulation such as migraine and excessive nocturnal hypotension are more common in the FI group than in the other ODP groups [5]. Furthermore, glaucomatous eyes with the FI phenotype show more rapid optic disc and VF progression compared to the other phenotypes [20]. In contrast, patients with MG ODP tend to be younger than those with FI ODP and are less likely to have systemic disorders [26]. However, to the best of our knowledge, there have been no reports describing differences in 24-h IOP patterns between FI and MG NTG patients, despite the fact that IOP is the most widely accepted risk factor for glaucoma pathogenesis [27–29]. Furthermore, knowledge of 24-h IOP patterns in NTG patients with different ODPs is helpful not only for understanding pathogenesis but also for prognosis and management of NTG patients. In the present study, there was a significant nocturnal elevation in habitual-position IOP in the FI group, whereas no such nocturnal IOP elevation was observed in the MG group matched for age and disease severity. Even though the MG group showed a significantly higher mean IOP during waking hours than the FI group, the FI group showed a higher mean IOP during sleeping hours and lower IOP nadir during the 24-h measurement period. Consequently, the magnitude of nocturnal IOP elevation was significantly greater in the FI group (P < 0.01). Indeed, FI ODP was an independent predictor of nocturnal IOP elevation in our multivariate GEE modeling.

While nocturnal IOP elevation may play a significant role in glaucoma pathogenesis [1–3, 30, 31], it is frequently undetected in clinical practice because routine IOP examinations are conducted during the daytime. Furthermore, glaucomatous VF progression has been associated with nocturnal events such as diminished nocturnal ocular perfusion pressure owing to low systemic blood pressure (BP) and/or nocturnal IOP elevation in the presence of normal daytime IOP [32–35]. Therefore, the current findings highlight the importance of detecting nocturnal IOP elevation and emphasize the clinical utility of 24-h habitual-position IOP measurements in NTG patients. In a previous study by Deokule et al [6], CE ODP was associated with higher nocturnal IOP compared to non-CE ODPs in POAG patients during 24-h IOP measurement. However, the mean daytime IOP readings were also greater in the CE group than the non-CE group, suggesting that the persistently elevated IOP in the eyes of CE ODP patients may be related to more severe structural ONH damage such as generalized neural rim loss and cupping. However, significant differences in 24-h IOP profiles among NTG patients with different ODPs had not been reported (e.g., FI vs. MG ODP).

In the current study, the mean daytime habitual-position IOP of the MG group was significantly higher than that of the FI group. In the recumbent position, however, the FI group showed higher mean nocturnal IOP than the MG group, with significantly greater nocturnal IOP elevation. One speculation for these findings is that postural change may have a stronger effect on nocturnal IOP in the FI group than in the MG group. Postural change may induce an elevation in episcleral venous pressure (EVP) due to increased choroidal volume and vascular engorgement [30, 36, 37], which may consequently elevate nocturnal IOP in the recumbent position as observed in the FI group. In contrast, myopic eyes as defined by RE are reported to have less choroidal volume than emmetropic eyes [38–41]. In addition, they have smaller collagen fiber bundles and individual collagen fibrils within the sclera, and reduced scleral thickness resulting in lower scleral rigidity [42, 43]. Although the MG group in the present study was defined according to optic disc appearance, mean RE was significantly more myopic (SE = −4.92 D) than that of the FI group (SE = −0.28D). As a result, less choroidal volume expansion at nighttime following posture change in the MG group may lead to reduced EVP. In addition, more “loose” ciliary body intercellular space and lower scleral rigidity in the MG group may result in greater uveoscleral outflow than in FI group patients at night [19, 37]. This combination of reduced EVP and increased uveoscleral outflow in the MG group may have suppressed IOP elevation at nighttime. These findings are in accord with our recent study on myopic glaucomatous-appearing patients of younger age (mean age = 32 years) and greater degree of myopia defined by RE (mean SE = −7 D) [19]. Future aqueous humor dynamic studies are needed to confirm our speculations.

Although postural changes may induce an elevation of EVP due to choroidal volume expansion and consequently raise recumbent-position nocturnal IOP as noted in the FI group [30, 36, 37], choroidal volume change may only partially explain the nocturnal IOP elevation. The effect of postural change on IOP elevation was measured after both short-term (10 min) and long-term (several hour) intervals. Choroidal expansion following posture change is expected to achieve equilibrium after long-term posture change. In the FI group, however, significant nocturnal IOP elevation persisted even after long-term posture change. This suggests that nocturnal IOP in FI group patients is not only enhanced by greater choroidal volume associated with posture change but by other factors that may affect ADHs. These factors may include axial length, baseline IOP level, angle anatomy, uveoscleral outflow, and CCT [19].

Our recent study found significant habitual-position IOP increases at nighttime in NTG eyes with a low baseline IOP, whereas no such nocturnal IOP elevation was noted in eyes with high baseline IOP [4]. Although the mechanism for this difference in nocturnal IOP response to postural changes between NTG patients with low and high baseline IOP remains elusive, we suggest it may be related to distinct EVP responses or to a combination of EVP and uveoscleral outflow rate differences between FI and MG groups at night [4,37]. In the present study, the FI group exhibited lower baseline IOP than the MG group (14.3 mmHg vs. 15.47 mmHg, P = 0.004) and no nocturnal IOP elevation was seen in the MG subjects with higher diurnal IOP levels. Therefore, the difference in baseline IOP between groups may also contribute to the difference in nocturnal IOP elevation between FI and MG groups.

Another possible explanation for our observations is that CCT has been shown to change over 24 h and influence 24-h hand-held tonometer readings [44, 45]. In other words, as CCT changes over 24 h, IOP readings may also change differentially between groups during the nocturnal period [46]. Despite similar baseline pachymetry values, 24-h variations in CCT due to distinct corneal biomechanical properties of MG and FI patients may result in different CCT values at nighttime that in turn give rise to different nocturnal IOP readings. Again, nocturnal IOP elevation may be affected by a variety of other ocular elements including corneal biomechanical changes over time.

In addition to mean IOP level, peak IOP during 24 h is considered to be a major risk factor for glaucoma [1, 47–50]. Therefore, it is important to know the time of peak IOP occurrence (acrophase) in glaucoma patients, as this can potentially influence the choice of medical therapy and the optimal dose. Of note, the most frequent peak IOP time differed between MG and FI groups. In the FI group, the peak IOP occurred most frequently during the night (12 PM–6 AM).

The magnitude of the nocturnal IOP peak in the FI group was similar to that reported previously in NTG eyes with similar RE [19]. By contrast, peak IOP in the MG group occurred most frequently during the morning hours (8 AM–12 PM). Although the physiological mechanism remains unclear, we suggest that different AHDs may result in distinct nocturnal IOP patterns between FI and MG ODPs.

The present study is unique in that we attempted to evaluate both overall acrophase according to ODP (FI vs. MG) and individual acrophases within the groups. Using least-squares cosinor rhythmometry analysis [2,4,19,20,23,24], the FI group showed highest mean IOP at nighttime (12 PM–6 AM) when patients were in the supine position. In individual analysis, three different IOP rhythms were identified among our 82 FI patients: nocturnal acrophase (54.9%), diurnal acrophase (12.2%), and no acrophase (32.9%). In contrast, analysis of the overall MG group showed no acrophase. In individual analysis, significantly different acrophase patterns were found in the MG group compared to the FI group: nocturnal acrophase (10.9%), diurnal acrophase (52.4%), and no identifiable acrophase (36.6%). The acrophase patterns found in each group paralleled the distributions of the most frequent peak times in each group as noted in Fig 2A & 2B. Since both age and disease severity are matched between groups, the physiologic mechanisms for the different acrophases must be related to other AHDs unaccounted for in the present study. Future studies on various ocular elements are needed to identify these factors.

Based on the results of our 24-h IOP analysis, nocturnal IOP elevation may play an important role in the pathogenesis of NTG with FI ODP. Recent reports including our own studies [32–34] have suggested that glaucoma progression is significantly associated with nocturnal events, such as low nocturnal blood pressure (BP) and elevated nocturnal IOP, in some NTG subjects having low to normal daytime IOP (IOP < 15 mm Hg). The FI group in the current study showed relatively low IOP (mean = 14 mm Hg) during office hours. However, these FI eyes with low diurnal IOP have greater nocturnal habitual-position IOP elevation, which can lead to glaucomatous disease progression due directly to the elevated IOP or to reduced ocular perfusion pressure in combination with reduced systemic BP at night.

In contrast to the FI group, the onset and progression of glaucoma in the MG group may be associated with persistently elevated IOP throughout 24 h (mean = 16mm Hg). POAG patients with MG and CE ODPs have been previously characterized as younger with fewer systemic risk factors for glaucoma [26]. Similar to POAG patients with CE ODP, NTG patients with MG ODP may have persistently elevated IOP (higher mean 24-h IOP compared to FI group) in the absence of other systemic risk factors. Therefore, this 24-h elevated IOP pattern may be closely linked to the underlying pathophysiology of glaucoma with MG ODP. However, NTG eyes with different ODPs should not be regarded as having different diseases as NTG is part of a continuum of OAG with different risk factors and presenting with different phenotypes.

The limitations of our study include the measurements of habitual-position IOP using a hand-held tonometer in the hospital (i.e., sitting during the day and supine at night), which may not provide the best physiological 24-h IOP data. In the future, the use of a continuous implantable IOP sensor in the habitual body position at home may provide more accurate measure of 24-h IOP data. Another possible limitation of our study is an inability to generalize our findings to patients with high-tension glaucoma (classified by an IOP level > 21 mm Hg) and to non-Asian individuals, as only Korean NTG patients were included in our current study. Further, relatively few IOP measurements were conducted (n = 11) during 24 h as these were performed manually, which can affect 24-h IOP rhythm [51]. Finally, ODP classification was ‘forced choices’ in some eyes with mixed appearance based on predominant features. Ideally, only pure ODPs should be selected. However, mixed disc appearance does occur, so the current study is more representative of the real clinical situation.

## Conclusion

A significant nocturnal IOP elevation was found in untreated NTG patients with FI ODP when IOP was measured in habitual positions, while MG ODP group showed no significant habitual-position IOP elevation at night. A cosinor model revealed statistically different 24-h acrophase patterns between FI and MG groups. Finally, FI ODP was an independent predictor of nocturnal IOP elevation in our series of untreated NTG patients.

## Supporting Information

S1 File Data set file. Total patients data of basic demographics, ophthalmologic examination, 24-hour intraocular pressure, blood pressure and ocular perfusion pressure.
(XLSX)
